# Commentary: Can a modernised psychiatric unit space reduce the use of coercive measures in child and adolescent psychiatry? A commentary on Czernine et al. (2024)

**DOI:** 10.1111/camh.12732

**Published:** 2024-08-16

**Authors:** Keri Ka‐Yee Wong

**Affiliations:** ^1^ Department of Psychology and Human Development University College London London UK; ^2^ UCL Centre for Education and Criminal Justice University College London London UK

**Keywords:** Environment, Child and adolescent psychiatry, Measurement, Participatory research

## Abstract

The environment influences the way we act, react and adapt to our surroundings whether it is consciously or unconsciously. Though it is widely accepted that multiple interacting systems influence human behaviour and development across the life span, the reality of teasing these factors apart is difficult and challenging. In this brief commentary on Czernine and colleagues' important and timely paper, ‘Can a modernised psychiatric unit space reduce the use of coercive measures in child and adolescent psychiatry?’, I evaluate and build on the evidence presented by making constructive suggestions on ways of improving the status quo healthcare and treatment conditions for children and adolescents today. The underlying assumption is that by furthering this complex yet important area of research in the field of psychiatry and adjacent disciplines, we can improve existing healthcare systems and processes that are aligned with meeting child and adolescent needs.

## Introduction

In any given environment, we, humans are exposed to a range of stimuli that influence our behaviour: from the smells in the space to the noise and temperature. We are all lived experience experts who act, react and adapt to our surroundings whether consciously or unconsciously. In fact, an individual's relationship with the environment has intrigued scientists alike – each discipline studying the factors in detail – yet it is widely accepted that multiple interacting layers of system influence human behaviour and development across the life span. For context, Bronfenbrenner's Ecological System's Theory (1979) is helpful as it underscores the complex interacting relationships between the individual and the wider systems in which we exist, including the *microsystem*, *mesosystem*, *exosystem* and *macrosystem*. Accepting that human behaviour and development are inextricably linked to the environment is not the challenge, but to tease apart the *cause* and *effect* of these layers of influence is difficult. In this paper, Czernine and colleagues asks (Czernine et al., 2024), ‘Can a modernised psychiatric unit space reduce the use of coercive measures in child and adolescent psychiatry?’

This important and timely issue highlights the use of mechanical coercive restraints in psychiatric settings – often considered a ‘necessary evil’ to control patients who are deemed to endanger oneself or others (National Collaborating centre for Mental Health, 2015) – whilst recognising the growing consensus in psychiatry that this practice is problematic. Not only is it a clear violation of patients' rights and should only be used as a last resort, like many others in the field of adult psychiatry, Czernine and colleagues call for alternative considerations of interventions (Czernine et al., 2024). This ‘rethinking’ of alternative interventions is especially vital in child and adolescent psychiatry where power imbalance between doctors and minors come into play, and where minors are often deemed vulnerable and without a say in treatment decisions. In their paper, Czernine and colleagues (Czernine et al., 2024) present a naturalistic observational study of one such alternative: that is a new hospital space as an ‘environmental intervention’. The authors ask the important directional question of whether a newly modernised psychiatric unit space can *reduce* the proportion, frequency and duration of mechanical coercive restraints on children and adolescents – the findings of which intersect multiple disciplines (i.e. psychiatry, architecture, psychology) and could inform guidance for clinicians and the development of alternative clinical and research practice that adds to the scant but growing current evidence in the field.

I commend the authors in bringing this topic to the fore, kick‐starting the field to reconsider ways to ultimately improve healthcare and treatment conditions for children and adolescents. Czernine and colleagues (Czernine et al., 2024) addressed this question by assigning patient records of 782 individuals into two groups and comparing independent groups on levels of coercive restraints and sample characteristics: those who were admitted into the hospital one and a half years *before* (*n* = 401) the move in October 2020 compared to those admitted *after* the move (*n* = 381). Whilst both groups did not differ on gender or psychiatric diagnosis – factors that have been found by previous studies to have an impact on coercive restraints – a few caveats should be discussed in turn that may attenuate the authors' conclusion that ‘improved hospital architecture can reduce the need for coercive measures’.

## Sources of information

The source of information for coercive restraints, according to the authors, are detailed official Austrian civil commitment law documentation for each patient's experience of coercive measures – since coercion and its uses are reported to the court and patient advocate promptly and regulated by the Austrian civil commitment law (Unterbringungsgesetz, UbG).^1^ This is a clear strength in the study as it offers a ‘near’ complete picture of patients that required extreme restrictive measures. However this number does not reflect whether the environmental changes in the new hospital caused the constraints in the first place The key ‘voices’ missing here are that of healthcare doctors and nursing staff. As coercive restraints are administered by healthcare professionals, an assessment of how and why coercive restraints were or were not administered would help corroborate objective measures of restraints on record. Of relevance to this paper is a participatory project Humanizing Hospitals (Morhayim & Wong, 2024), where my colleagues and I coproduced a toolkit with cancer patients to understand the role of the hospital treatment infusion units on patient's mental and physical health. Such tools recognise the importance of patient perspectives and can assist doctors, architects and researchers on future research questions. Thus, collating multiple informants would help identify more precisely which aspects of the hospital environment were conducive to fewer coercive restraints. Staff perception of the old and new hospitals is a confounder because it impacts both the independent variable (i.e. hospital) and dependent variable (i.e. coercion rates). Since the paper does not measure this, we cannot conclude definitively that the new hospital space directly caused the reductions in coercion. This relationship could be driven by staff generally feeling more positive about the new space, rather than the new features of the hospital specifically. Hypothetically, if staff were made to feel more positive in the old hospital (e.g. given time off to de‐stress or have a pay rise), might we see the same reductions in coercion rates? Maybe, maybe not. If we built the exact hospital elsewhere in the world, would we see reduced coercion rates even if the staff are still stressed and overworked? Thus, in the current paper, I think the experience of both the old and new hospital space – the change in how the environment has made the staff feel – may have contributed to the reduced coercion behaviour. But unless we have staff's attitudes, we cannot say that the ‘new hospital in itself reduced coercion rates’. Complementing official report data with healthcare staff's perspective of the hospital space may provide a useful follow‐up to the current study and a more nuanced picture of the environmental influences on coercive restraints in psychiatric settings.

## Research design

Another important point to recognise is that the significant group differences in coercive restraints attributed to the new hospital environment alone is problematic given that comparisons are made *between*‐individuals rather than *within*‐individuals. To give the current analyses more weight and recognising the limitations of naturalistic observational studies (e.g. lack of comparison group precludes manipulation of a single key variable), testing an *alternative null hypothesis* using the official patient record data may be helpful. By comparing the same outcome metrics (i.e. mechanical restraints, rate of seclusion, duration of coercion and proportion to length of stay) using the old hospital data ‘one and half years’ before 1 April 2019 of the first period of data included in the current study, it may be possible to test whether there are significant changes to the same metrics – despite the key difference being that there was no ‘intervention’ (hospital move). One may hypothesise that the metrics will be ‘nonsignificant’ as no intervention has taken place – but any differences can be attributed to temporal changes that would happen naturally. Although this is not a perfect comparison based on administrative data – testing a counterfactual null hypothesis can strengthen the study conclusions. Although the ideal research design would be to examine two groups of patients who did not differ on characteristics but remained in the old hospital versus a second group of patients who had moved into the new hospital over the same duration period, this is not a feasible design option and thus support for a cause (new hospital) and effect (reduced coercion) model is reduced.

## Who should have a say?

Finally, against the backdrop of clear strengths and opportunities that this naturalistic observational study has yielded (e.g. prospective design, evenly distributed diagnosis, large sample size, specificity of coercive measures), one of the most powerful yet absent ‘voices’ that should also be brought to the fore are the young children and adolescents who are in these spaces. By taking a child‐centred participatory approach on the topic, we ensure that our healthcare systems and practitioners have children and adolescent's best interest. It is important that we are working with children for children and that lived experience experts are part of our research agenda, helping us shape our rethinking and development of treatments and ensure that there is a safe open space for continued discussions around care. For example, my own participatory research on the impact of COVID‐19 on young people's mental health, physical health and social relationships (Wong & Raine, 2020) have highlighted disproportionate impacts on specific groups of individuals that would be ‘camouflaged’ if only group averages were reported. Instead, my lab and colleagues around the world have highlighted the disproportionate impacts that the COVID‐19 pandemic has had on specific groups of young people, including minority ethnic young children and adolescents around the world (Creswell, 2023; Lee & Wong, 2024; Lenoir & Wong, 2023), as these groups are seldom included in decisions. Through giving voice to young children in research, we are representing them in our data, which is the only way to ensure that decision‐makers and policymakers have a chance of understanding what the real needs are on the ground. Building on Czernine and colleague's study (Czernine et al., 2024), perhaps an opportunity exists to ask the children and young patients in the hospital: ‘what does a supportive treatment environment focused on enhancing your recovery and mental wellbeing look like?’

## Concluding remarks

In sum, the environment, and the spaces we occupy evidently have a role to play in our mood, health and behaviours. The study of the role of the built environment – particularly hospital spaces where patients stay for treatment of ill‐health is of utmost importance as it can directly *impact* – or at the very least – *moderate* patient's health and healthcare professionals' treatment behaviours. It remains extremely difficult to precisely tease apart the relative contributions of the environment on human behaviour (granted the COVID‐19 pandemic afforded a ‘naturalistic experiment’ to study this question in more detail that normally is not possible) (Wong, 2022), but Czernine and colleague's study certainly makes this first important contribution in the right direction. I wholeheartedly agree with the authors that ‘adequate and child‐friendly architectural structures are an important factor in ensuring best care for children and adolescents in mental health crises’, and would encourage future follow‐up studies, replications and hypothesis testing to continue – something that a newly special issue at ACAMH is currently addressing (Physical Environmental Influences on the Psychosocial Outcomes of Children, Adolescents and Young Adults). Through participatory approaches and codesign, complemented by qualitative feedback from healthcare professionals, and involvement from child and adolescent lived experience experts, I believe the field will be well‐placed to eventually inform the development of a new assessment tool that begins to yield valuable insights into *which aspects* and *how* hospital spaces are related to reductions in coercion and patient health outcomes overtime.

## Ethical information

No ethical approval was required for this article.

## Footnotes

^1^
The interests of those affected are protected in particular by existing representations for these areas: the patient advocacy in the Hospitalisation Act and the residents' representation in the Nursing Home Residence; in detail Kopetzki (2012), Strickmann (2012), Ganner (2017). Cited from source: https://julgar.pt/wp‐content/uploads/2020/05/JULGAR41‐08‐MG.pdf on 18 March 2024.
